# 2-Amino-3-methyl­pyridinium 2-amino-5-methyl­pyridinium sulfate monohydrate

**DOI:** 10.1107/S1600536809049241

**Published:** 2009-11-21

**Authors:** Jiang Gong, Gang Chen, Shi-Feng Ni, Yong-Yao Zhang, Hai-Bin Wang

**Affiliations:** a Department of Medicine, Tibet Nationalities Institute, Xianyang, Shaanxi 712082, People’s Republic of China; b Key Laboratory of Resource Biology and Biotechnology in Western China, Ministry of Education, College of Life Science, Northwest University, Xi’an 710069, People’s Republic of China; c College of Pharmaceutical Sciences, Zhejiang University of Technology, Hangzhou 310014, People’s Republic of China; d College of Chemical Engineering and Materials Science, Zhejiang University of Technology, Hangzhou 310014, People’s Republic of China

## Abstract

The asymmetric unit of the title compound, 2C_6_H_9_N_2_^+^·SO_4_^2−^·H_2_O, contains two isomeric protonated amino­methyl­pyridine cations, a sulfate anion and a solvent water mol­ecule. The cations are in the iminium tautomeric form. In the crystal structure, inter­molecular O—H⋯O, N—H⋯O and weak C—H⋯O hydrogen bonds link the components into a three-dimensional network. Additional stabilization is provided by weak π–π stacking inter­actions, with centroid–centroid distances of 3.758 (2) and 3.774 (1) Å.

## Related literature

For related structures, see: Nahringbauer & Kvick (1977 ▶); Espenbetov *et al.* (1985 ▶); Jin *et al.* (2000 ▶, 2001 ▶, 2005 ▶); Luque *et al.* (1997 ▶). For studies on the tautomeric forms of 2-amino­pyridine systems, see: Inuzuka & Fujimoto (1986 ▶, 1990 ▶); Ishikawa *et al.* (2002 ▶).
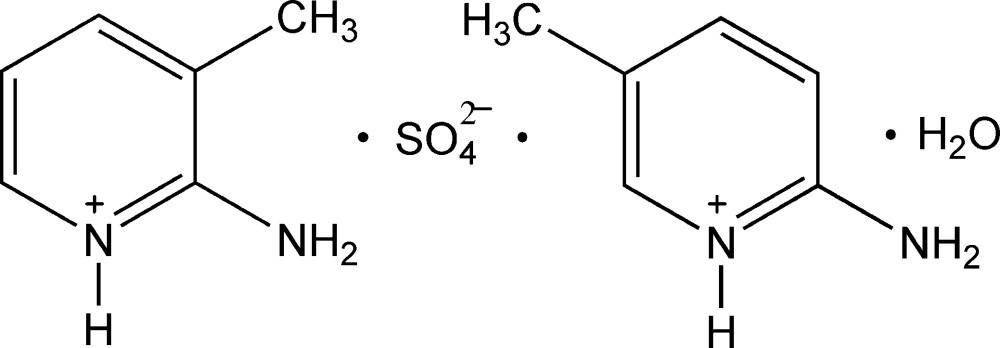


## Experimental

### 

#### Crystal data

2C_6_H_9_N_2_^+^·SO_4_^2−^·H_2_O
*M* *_r_* = 332.39
Monoclinic, 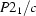

*a* = 8.4071 (7) Å
*b* = 20.7654 (17) Å
*c* = 9.3369 (8) Å
β = 103.983 (1)°
*V* = 1581.7 (2) Å^3^
*Z* = 4
Mo *K*α radiation
μ = 0.23 mm^−1^
*T* = 293 K
0.30 × 0.30 × 0.30 mm

#### Data collection

Bruker SMART APEX area-detector diffractometer
Absorption correction: multi-scan (*SADABS*; Bruker, 2000 ▶) *T*_min_ = 0.908, *T*_max_ = 0.923
8087 measured reflections
2780 independent reflections
2492 reflections with *I* > 2σ(*I*)
*R*_int_ = 0.015

#### Refinement

*R*[*F* ^2^ > 2σ(*F* ^2^)] = 0.043
*wR*(*F* ^2^) = 0.129
*S* = 1.06
2780 reflections
207 parameters
H atoms treated by a mixture of independent and constrained refinement
Δρ_max_ = 0.37 e Å^−3^
Δρ_min_ = −0.38 e Å^−3^

### 

Data collection: *SMART* (Bruker, 2000 ▶); cell refinement: *SAINT* (Bruker, 2000 ▶); data reduction: *SAINT*; program(s) used to solve structure: *SHELXS97* (Sheldrick, 2008 ▶); program(s) used to refine structure: *SHELXL97* (Sheldrick, 2008 ▶); molecular graphics: *SHELXTL* (Sheldrick, 2008 ▶); software used to prepare material for publication: *SHELXL97*.

## Supplementary Material

Crystal structure: contains datablocks global, I. DOI: 10.1107/S1600536809049241/lh2943sup1.cif

Structure factors: contains datablocks I. DOI: 10.1107/S1600536809049241/lh2943Isup2.hkl

Additional supplementary materials:  crystallographic information; 3D view; checkCIF report

## Figures and Tables

**Table 1:** Hydrogen-bond geometry (Å, °)

*D*—H⋯*A*	*D*—H	H⋯*A*	*D*⋯*A*	*D*—H⋯*A*
N1—H1⋯O1	0.86	1.82	2.657 (2)	164
N2—H2*A*⋯O2	0.86	2.14	2.991 (3)	170
N3—H3⋯O3	0.86	1.93	2.781 (3)	173
N4—H4*A*⋯O1	0.86	2.02	2.826 (3)	156
N4—H4*B*⋯O5	0.86	2.07	2.857 (3)	152
C5—H5⋯O5	0.93	2.41	3.334 (3)	171
O5—H5*B*⋯O2^i^	0.82 (3)	2.03 (3)	2.833 (3)	167 (3)
O5—H5*A*⋯O3^ii^	0.80 (2)	2.10 (4)	2.845 (3)	157 (3)
C2—H2⋯O1^iii^	0.93	2.41	3.334 (3)	176
N2—H2*B*⋯O4^iii^	0.86	1.99	2.835 (3)	168
C11—H11⋯O3^iv^	0.93	2.56	3.317 (3)	138

Symmetry codes: (i) 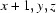; (ii) 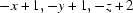; (iii) 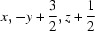; (iv) 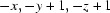.

**Fig. 1.**
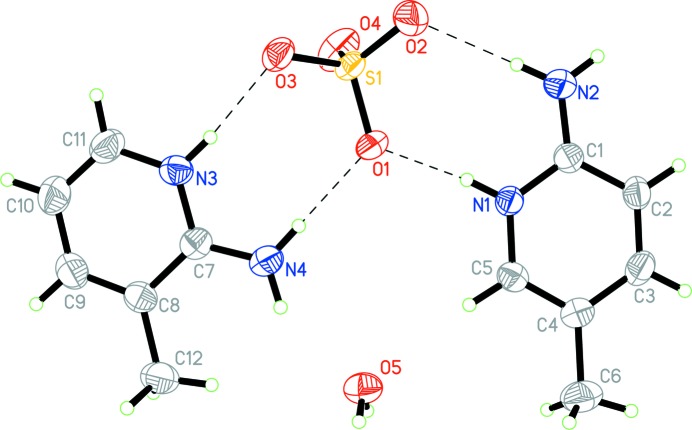
The asymmetric unit of (I) showing 40% probabilty ellipsoids for non-hydrogen atoms. The dashed lines indicate hydrogen bonds.

**Fig. 2.**
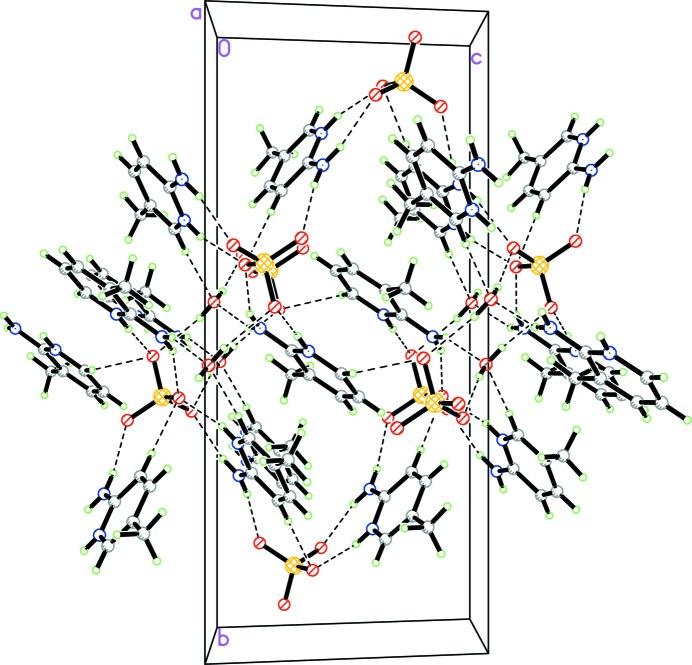
Part of the crystal structure showing hydrogen bonds as dashed lines.

## supplementary crystallographic information

### Comment

We are not aware of any articles which report crystal structures containing different two pyridininium cations and a sulfate cation. We present the crystal structure of the title compound, (I), herein.

The asymmetric unit of the title compound (I) is shown in Fig. 1. Protonation of atom N1 of the 2-amino-5-methyl-pyridine and N3 of 2-amino-3-methyl-pyridine cation results in a widening of the C1—N1—C5 and C7—N3—C11 angles. These values can be compared to those of 117.5 (3)° in neutral 2-amino-5-methyl-pyridine (Nahringbauer & Kvick, 1977) and 118.0 (2)° in neutral 2-amino-3-methyl-pyridine (Espenbetov *et al.*, 1985). The C1-C5/N1 ring and C7-C11/N3 pyridinium rings are both essentially planar, with a maximum deviation from the mean plane of the rings of 0.024 (3)Å for atom N2 and 0.007 (3)Å for atom C9. The geometries of the two pyridinium rings are similar to those observed in other 2-aminopyridine structures (Luque *et al.*, 1997; Jin *et al.*, 2000,2001,2005) that are in the iminium tautomeric form (Inuzuka & Fujimoto, 1986,1990; Ishikawa *et al.*, 2002).

In the crystal structure, intermolecular O-H···O, N-H···O and weak C-H···O hydrogen bonds link the components of the structure into a three-dimensional network (Fig. 2). Additional stabilization is provided by weak π–π stacking interactions with centroid to centroid distances of 3.758 (2) and 3.774 (1)Å.

### Experimental

2-Amino-3-methyl-pyridine, 2-amino-5-methyl-pyridine and sulfuric acid were mixed in molar ratio 1:1:1 and dissolved in sufficient water. The solution was stirred and heated until a clear solution resulted. Colourless crystals of (I) were formed by gradual evaporation of excess water over a period of one week at 293 K.

### Refinement

H atoms of the water molecule were located in a differnce Fourier map, and were refined independently with isotropic displacement parameters. Other H atom were placed in calculated positions and allowed to ride on their parent atoms at distances of 0.93 Å for aromatic C atoms, 0.86 Å for amido and 0.96 Å for methyl with isotropic displacement parameters 1.2 times *U*_eq_ of the parent atoms or 1.5 times U_eq_ for methyl C atoms.

### Crystal data

**Table:**

2C_6_H_9_N_2_^+^·SO_4_^2^^−^·H_2_O	*Z* = 4
*M**_r_* = 332.39	*F*(000) = 704.0
Monoclinic, *P*2_1_/*c*	*D*_x_ = 1.396 Mg m^−^^3^
Hall symbol: -P 2ybc	Mo *K*α radiation, λ = 0.71073 Å
*a* = 8.4071 (7) Å	θ = 2.1–25.1°
*b* = 20.7654 (17) Å	µ = 0.23 mm^−^^1^
*c* = 9.3369 (8) Å	*T* = 293 K
β = 103.983 (1)°	Prism, colorless
*V* = 1581.7 (2) Å^3^	0.30 × 0.30 × 0.30 mm

### Data collection

**Table:**

Bruker SMART APEX area-detector diffractometer	2780 independent reflections
Radiation source: fine-focus sealed tube	2492 reflections with *I* > 2σ(*I*)
graphite	*R*_int_ = 0.015
φ and ω scan	θ_max_ = 25.0°, θ_min_ = 2.0°
Absorption correction: multi-scan (*SADABS*; Bruker, 2000)	*h* = −10→9
*T*_min_ = 0.908, *T*_max_ = 0.923	*k* = −24→23
8087 measured reflections	*l* = −11→10

### Refinement

**Table:**

Refinement on *F*^2^	Primary atom site location: structure-invariant direct methods
Least-squares matrix: full	Secondary atom site location: difference Fourier map
*R*[*F*^2^ > 2σ(*F*^2^)] = 0.043	Hydrogen site location: inferred from neighbouring sites
*wR*(*F*^2^) = 0.129	H atoms treated by a mixture of independent and constrained refinement
*S* = 1.06	*w* = 1/[σ^2^(*F*_o_^2^) + (0.0672*P*)^2^ + 1.0523*P*] where *P* = (*F*_o_^2^ + 2*F*_c_^2^)/3
2780 reflections	(Δ/σ)_max_ = 0.001
207 parameters	Δρ_max_ = 0.37 e Å^−^^3^
0 restraints	Δρ_min_ = −0.37 e Å^−^^3^

### Special details

**Table:**

Geometry. All e.s.d.'s (except the e.s.d. in the dihedral angle between two l.s. planes) are estimated using the full covariance matrix. The cell e.s.d.'s are taken into account individually in the estimation of e.s.d.'s in distances, angles and torsion angles; correlations between e.s.d.'s in cell parameters are only used when they are defined by crystal symmetry. An approximate (isotropic) treatment of cell e.s.d.'s is used for estimating e.s.d.'s involving l.s. planes.
Refinement. Refinement of *F*^2^ against ALL reflections. The weighted *R*-factor *wR* and goodness of fit *S* are based on *F*^2^, conventional *R*-factors *R* are based on *F*, with *F* set to zero for negative *F*^2^. The threshold expression of *F*^2^ > σ(*F*^2^) is used only for calculating *R*-factors(gt) *etc*. and is not relevant to the choice of reflections for refinement. *R*-factors based on *F*^2^ are statistically about twice as large as those based on *F*, and *R*- factors based on ALL data will be even larger.

### Fractional atomic coordinates and isotropic or equivalent isotropic displacement parameters (Å^2^)

**Table:**

	*x*	*y*	*z*	*U*_iso_*/*U*_eq_	
H5B	0.897 (4)	0.5692 (16)	0.975 (4)	0.072 (10)*	
H5A	0.873 (4)	0.5243 (17)	1.060 (4)	0.076 (12)*	
S1	0.13278 (6)	0.60692 (2)	0.79985 (6)	0.03698 (19)	
O1	0.3104 (2)	0.60658 (8)	0.8712 (2)	0.0532 (5)	
O2	0.0451 (2)	0.63253 (9)	0.9043 (2)	0.0589 (5)	
O3	0.08256 (19)	0.53962 (8)	0.76110 (18)	0.0468 (4)	
O4	0.1027 (3)	0.64557 (10)	0.6672 (2)	0.0701 (6)	
O5	0.8248 (2)	0.55071 (10)	1.0044 (2)	0.0524 (5)	
N1	0.4680 (2)	0.67850 (8)	1.09636 (19)	0.0366 (4)	
H1	0.4085	0.6519	1.0356	0.044*	
N2	0.2340 (2)	0.73695 (10)	1.0919 (2)	0.0537 (5)	
H2A	0.1785	0.7101	1.0292	0.064*	
H2B	0.1856	0.7691	1.1213	0.064*	
N3	0.3036 (2)	0.47027 (10)	0.6431 (2)	0.0439 (5)	
H3	0.2413	0.4940	0.6818	0.053*	
N4	0.5202 (3)	0.50690 (11)	0.8229 (2)	0.0550 (6)	
H4A	0.4523	0.5294	0.8577	0.066*	
H4B	0.6232	0.5080	0.8647	0.066*	
C1	0.3949 (3)	0.72881 (11)	1.1434 (2)	0.0383 (5)	
C2	0.4942 (3)	0.77118 (11)	1.2445 (3)	0.0452 (5)	
H2	0.4480	0.8064	1.2807	0.054*	
C3	0.6580 (3)	0.76018 (12)	1.2886 (3)	0.0489 (6)	
H3A	0.7229	0.7884	1.3552	0.059*	
C4	0.7325 (3)	0.70730 (12)	1.2365 (3)	0.0443 (5)	
C5	0.6321 (3)	0.66757 (11)	1.1403 (2)	0.0406 (5)	
H5	0.6764	0.6320	1.1035	0.049*	
C6	0.9147 (3)	0.69585 (16)	1.2847 (4)	0.0686 (8)	
H6A	0.9644	0.7290	1.3526	0.103*	
H6B	0.9607	0.6966	1.2001	0.103*	
H6C	0.9350	0.6546	1.3322	0.103*	
C7	0.4663 (3)	0.47068 (11)	0.7055 (2)	0.0409 (5)	
C8	0.5701 (3)	0.43135 (11)	0.6419 (3)	0.0443 (5)	
C9	0.4968 (4)	0.39642 (13)	0.5209 (3)	0.0584 (7)	
H9	0.5618	0.3708	0.4764	0.070*	
C10	0.3270 (4)	0.39747 (14)	0.4606 (3)	0.0668 (8)	
H10	0.2803	0.3727	0.3783	0.080*	
C11	0.2333 (3)	0.43492 (13)	0.5239 (3)	0.0553 (7)	
H11	0.1205	0.4364	0.4856	0.066*	
C12	0.7509 (3)	0.42992 (14)	0.7089 (3)	0.0584 (7)	
H12A	0.7763	0.4577	0.7935	0.088*	
H12B	0.8083	0.4444	0.6376	0.088*	
H12C	0.7840	0.3867	0.7385	0.088*	

### Atomic displacement parameters (Å^2^)

**Table:**

	*U*^11^	*U*^22^	*U*^33^	*U*^12^	*U*^13^	*U*^23^
S1	0.0324 (3)	0.0332 (3)	0.0414 (3)	−0.0057 (2)	0.0014 (2)	0.0011 (2)
O1	0.0341 (9)	0.0525 (10)	0.0654 (11)	−0.0031 (7)	−0.0027 (8)	−0.0152 (8)
O2	0.0513 (11)	0.0522 (11)	0.0765 (12)	−0.0050 (8)	0.0215 (9)	−0.0146 (9)
O3	0.0433 (9)	0.0382 (9)	0.0543 (10)	−0.0094 (7)	0.0030 (7)	−0.0033 (7)
O4	0.0798 (14)	0.0605 (12)	0.0590 (12)	−0.0227 (10)	−0.0047 (10)	0.0196 (9)
O5	0.0385 (10)	0.0571 (11)	0.0607 (12)	−0.0011 (9)	0.0103 (9)	0.0047 (9)
N1	0.0374 (10)	0.0344 (9)	0.0361 (9)	−0.0005 (7)	0.0052 (7)	−0.0023 (7)
N2	0.0389 (11)	0.0536 (12)	0.0639 (14)	0.0080 (9)	0.0034 (10)	−0.0141 (10)
N3	0.0387 (10)	0.0449 (11)	0.0465 (11)	0.0045 (8)	0.0071 (8)	0.0032 (9)
N4	0.0383 (11)	0.0714 (15)	0.0506 (12)	0.0024 (10)	0.0018 (9)	−0.0153 (11)
C1	0.0404 (12)	0.0390 (12)	0.0347 (11)	0.0041 (9)	0.0077 (9)	0.0022 (9)
C2	0.0516 (14)	0.0408 (12)	0.0415 (12)	0.0046 (10)	0.0080 (10)	−0.0081 (10)
C3	0.0511 (14)	0.0475 (14)	0.0425 (13)	−0.0055 (11)	0.0003 (11)	−0.0078 (10)
C4	0.0385 (12)	0.0476 (13)	0.0444 (12)	−0.0003 (10)	0.0052 (10)	0.0027 (10)
C5	0.0401 (12)	0.0391 (12)	0.0429 (12)	0.0053 (9)	0.0108 (10)	0.0018 (9)
C6	0.0398 (14)	0.077 (2)	0.083 (2)	−0.0004 (13)	0.0031 (13)	−0.0047 (17)
C7	0.0430 (12)	0.0402 (12)	0.0375 (11)	−0.0011 (9)	0.0060 (9)	0.0052 (9)
C8	0.0453 (13)	0.0412 (12)	0.0459 (12)	0.0055 (10)	0.0101 (10)	0.0056 (10)
C9	0.0624 (17)	0.0525 (15)	0.0592 (16)	0.0093 (12)	0.0127 (13)	−0.0087 (12)
C10	0.0681 (19)	0.0652 (18)	0.0580 (17)	0.0031 (14)	−0.0028 (14)	−0.0178 (14)
C11	0.0488 (14)	0.0547 (15)	0.0529 (15)	−0.0019 (12)	−0.0061 (12)	−0.0008 (12)
C12	0.0462 (14)	0.0590 (16)	0.0687 (17)	0.0090 (12)	0.0116 (13)	0.0029 (13)

### Geometric parameters (Å, °)

**Table:**

S1—O4	1.4460 (19)	C2—H2	0.9300
S1—O2	1.4577 (19)	C3—C4	1.408 (3)
S1—O3	1.4792 (16)	C3—H3A	0.9300
S1—O1	1.4808 (17)	C4—C5	1.354 (3)
O5—H5B	0.82 (4)	C4—C6	1.508 (3)
O5—H5A	0.79 (4)	C5—H5	0.9300
N1—C1	1.339 (3)	C6—H6A	0.9600
N1—C5	1.360 (3)	C6—H6B	0.9600
N1—H1	0.8600	C6—H6C	0.9600
N2—C1	1.333 (3)	C7—C8	1.426 (3)
N2—H2A	0.8600	C8—C9	1.358 (4)
N2—H2B	0.8600	C8—C12	1.498 (3)
N3—C11	1.345 (3)	C9—C10	1.402 (4)
N3—C7	1.351 (3)	C9—H9	0.9300
N3—H3	0.8600	C10—C11	1.341 (4)
N4—C7	1.316 (3)	C10—H10	0.9300
N4—H4A	0.8600	C11—H11	0.9300
N4—H4B	0.8600	C12—H12A	0.9600
C1—C2	1.407 (3)	C12—H12B	0.9600
C2—C3	1.358 (3)	C12—H12C	0.9600
			
O4—S1—O2	111.00 (13)	C4—C5—N1	121.5 (2)
O4—S1—O3	109.53 (11)	C4—C5—H5	119.2
O2—S1—O3	110.35 (10)	N1—C5—H5	119.2
O4—S1—O1	109.70 (12)	C4—C6—H6A	109.5
O2—S1—O1	108.56 (11)	C4—C6—H6B	109.5
O3—S1—O1	107.63 (9)	H6A—C6—H6B	109.5
H5B—O5—H5A	104 (3)	C4—C6—H6C	109.5
C1—N1—C5	122.93 (19)	H6A—C6—H6C	109.5
C1—N1—H1	118.5	H6B—C6—H6C	109.5
C5—N1—H1	118.5	N4—C7—N3	118.1 (2)
C1—N2—H2A	120.0	N4—C7—C8	123.5 (2)
C1—N2—H2B	120.0	N3—C7—C8	118.3 (2)
H2A—N2—H2B	120.0	C9—C8—C7	116.9 (2)
C11—N3—C7	123.8 (2)	C9—C8—C12	123.2 (2)
C11—N3—H3	118.1	C7—C8—C12	119.9 (2)
C7—N3—H3	118.1	C8—C9—C10	122.6 (3)
C7—N4—H4A	120.0	C8—C9—H9	118.7
C7—N4—H4B	120.0	C10—C9—H9	118.7
H4A—N4—H4B	120.0	C11—C10—C9	118.8 (3)
N2—C1—N1	119.1 (2)	C11—C10—H10	120.6
N2—C1—C2	123.3 (2)	C9—C10—H10	120.6
N1—C1—C2	117.6 (2)	C10—C11—N3	119.6 (2)
C3—C2—C1	119.5 (2)	C10—C11—H11	120.2
C3—C2—H2	120.3	N3—C11—H11	120.2
C1—C2—H2	120.3	C8—C12—H12A	109.5
C2—C3—C4	122.0 (2)	C8—C12—H12B	109.5
C2—C3—H3A	119.0	H12A—C12—H12B	109.5
C4—C3—H3A	119.0	C8—C12—H12C	109.5
C5—C4—C3	116.5 (2)	H12A—C12—H12C	109.5
C5—C4—C6	121.9 (2)	H12B—C12—H12C	109.5
C3—C4—C6	121.6 (2)		
			
C5—N1—C1—N2	178.2 (2)	C11—N3—C7—C8	−0.1 (3)
C5—N1—C1—C2	−0.9 (3)	N4—C7—C8—C9	179.8 (2)
N2—C1—C2—C3	−178.4 (2)	N3—C7—C8—C9	0.6 (3)
N1—C1—C2—C3	0.7 (3)	N4—C7—C8—C12	−0.1 (4)
C1—C2—C3—C4	−0.1 (4)	N3—C7—C8—C12	−179.3 (2)
C2—C3—C4—C5	−0.3 (4)	C7—C8—C9—C10	−0.9 (4)
C2—C3—C4—C6	179.3 (2)	C12—C8—C9—C10	179.0 (3)
C3—C4—C5—N1	0.2 (3)	C8—C9—C10—C11	0.7 (5)
C6—C4—C5—N1	−179.5 (2)	C9—C10—C11—N3	−0.1 (4)
C1—N1—C5—C4	0.4 (3)	C7—N3—C11—C10	−0.1 (4)
C11—N3—C7—N4	−179.4 (2)		

### Hydrogen-bond geometry (Å, °)

**Table:**

*D*—H···*A*	*D*—H	H···*A*	*D*···*A*	*D*—H···*A*
N1—H1···O1	0.86	1.82	2.657 (2)	164
N2—H2A···O2	0.86	2.14	2.991 (3)	170
N3—H3···O3	0.86	1.93	2.781 (3)	173
N4—H4A···O1	0.86	2.02	2.826 (3)	156
N4—H4B···O5	0.86	2.07	2.857 (3)	152
C5—H5···O5	0.93	2.41	3.334 (3)	171
O5—H5B···O2^i^	0.82 (3)	2.03 (3)	2.833 (3)	167 (3)
O5—H5A···O3^ii^	0.80 (2)	2.10 (4)	2.845 (3)	157 (3)
C2—H2···O1^iii^	0.93	2.41	3.334 (3)	176
N2—H2B···O4^iii^	0.86	1.99	2.835 (3)	168
C11—H11···O3^iv^	0.93	2.56	3.317 (3)	138

Symmetry codes: (i) *x*+1, *y*, *z*; (ii) −*x*+1, −*y*+1, −*z*+2; (iii) *x*, −*y*+3/2, *z*+1/2; (iv) −*x*, −*y*+1, −*z*+1.
